# Uncovering the molecular and immunological defects in multicentric carpotarsal osteolysis syndrome: identification of relevant biomarkers

**DOI:** 10.3389/fimmu.2025.1543385

**Published:** 2025-10-29

**Authors:** Dorra Najjar, Asma Chikhaoui, Rim Boussetta, Saifeddine Azouz, Sinda Zarrouk, Sami Bouchoucha, Houda Yacoub-Youssef

**Affiliations:** ^1^ Laboratory of Biomedical Genomics and Oncogenetics, LR16IPT05, Institut Pasteur de Tunis, Tunis El Manar University, Tunis, Tunisia; ^2^ Department of Pediatric Orthopedic Surgery, Bechir Hamza Children’s Hospital, Faculty of Medicine of Tunis, Tunis El Manar University, Tunis, Tunisia; ^3^ Genomics Platform, Institut Pasteur de Tunis, Tunis El Manar University, Tunis, Tunisia

**Keywords:** multicentric carpotarsal osteolysis, juvenile idiopathic arthritis, bone osteolysis, inflammation, non-steroid treatment, *MAFB* gene, nephropathy

## Abstract

Multicentric carpotarsal osteolysis (MCTO) is a rare genetic disease characterized by progressive osteolysis, often followed by nephropathy in advanced stages. It’s caused by variants in the *MAFB* gene. This disease mimics juvenile idiopathic arthritis (JIA) and is often misdiagnosed due to the clinical similarity and rarity of MCTO disease. However, the pathophysiology of MCTO remains largely unknown. While the use of non-steroidal treatment is recommended for patients with JIA, it triggers the onset of nephropathy in patients with MCTO. In this study, we aimed to investigate the clinical, genetic etiology, and immune profiles of patients with MCTO in three Tunisian siblings with MCTO disease. Genetic investigation was performed using Sanger sequencing, and the effect of the variant on the phosphorylation process was explored using the in-silico prediction tool “NetPhos”. We further investigated the expression of 17 immune-related genes using qPCR, and performed immune cell phenotyping using flow cytometry in these patients and in five healthy donors. Twelve inflammatory cytokines were measured using ELISArray. We observed clinical variability among the siblings, and a kidney biopsy revealed focal segmental glomerulosclerosis in one patient. The genetic analysis identified a novel variant in *MAFB* (c.187C>T; p.(Pro63Ser)) in the three patients, for whom in silico investigation revealed that this variant could lead to alterations in the phosphorylation process. Further investigations revealed that MCTO patients tend to have increased frequencies of non-classical and intermediate monocytes, which may be associated with bone osteolysis. Interestingly, high levels of CD8+ T cells, NK CD56bright cells, and IL8 were detected in a single patient who presented an early-stage nephropathy, which may be a consequence of the use of non-steroidal treatment. Inflammatory and oxidative stress-related genes were overexpressed. In conclusion, we present the first study on Tunisian patients in which the genetic investigation oriented the diagnosis from JIA to MCTO through the identification of a novel variant that affects the phosphorylation of the MafB protein. We suggest that both genetic and immune alterations may contribute to the development of MCTO syndrome and provide preliminary insights into its pathophysiology.

## 1 Introduction

Multicentric carpotarsal osteolysis with or without nephropathy (MCTO) (OMIM #166300) is a rare genetic disease characterized mainly by progressive osteolysis of the carpal and tarsal bones. In some cases, large joints, such as those in the elbow and knee, can be affected, leading to functional impairment and deformities. This disease is often confused with juvenile idiopathic arthritis (JIA) because of overlapping clinical features (1).

*De novo* or autosomal dominant inherited variants in the transactivation domain (residues 54–71) of the *MAFB* gene (MAF BZIP Transcription Factor B) are associated with MCTO disorder (2). To date, 69 patients have been reported and genetically confirmed (1–17). This region is susceptible to post-translational modifications. It presents four serine and one threonine residue that are phosphorylation target sites. Interestingly, the variants associated with MCTO disease are mostly located at these phosphorylation sites, targeted by the glycogen synthase kinase-3 protein (GSK3), which affects the stability and function of the MafB protein (18).

The *MAFB* gene is a transcription factor expressed in multiple tissues and cell types that plays multiple roles. It is involved in different biological processes, such as chondrocyte matrix formation; differentiation of α and β pancreatic cells, lymphangiogenesis, and regulation of IFN production. Its main function is the polarization of monocytes and macrophages into anti-inflammatory profiles (M2) and the inhibition of their differentiation into osteoclasts via the inhibition of the Receptor Activator of Nuclear Factor Kappa-B Ligand (RANKL)-induced mechanism (19). Recently, excessive osteoclast activity has been shown to be the causative mechanism of bone osteolysis in MCTO patients (18). The osteoclasts are one of the major cells involved in bone remodeling throughout life. The dysregulation of their differentiation or activity causes various bone diseases, ranging from osteopetrosis to osteoporosis, including inflammatory diseases such as Rheumatoid Arthritis (RA). Osteoclasts contribute to the inflammatory processes underlying these diseases, exacerbating bone osteolysis. Recently, new evidence has shown that osteoclasts play important roles in modulating the immune system toward inflammation or immune suppression. On the other hand, both innate and adaptive immune cells play important roles in controlling osteoclastogenesis (20). In fact, inflammatory cytokines and chemokines may promote ROS generation. Recent research has indicated that oxidative stress can impact osteoclast development and proliferation, resulting in an imbalance between osteoclast and osteoblast activities, which can lead to metabolic bone and skeletal disorders (21). However, their implications in rare skeletal disorders such as MCTO have never been investigated.

Despite the clinical overlap between MCTO and JIA, the use of non-steroidal anti-inflammatory drugs for JIA patients is harmful to those with MCTO due to the increased risk of developing nephropathy. Most patients with MCTO frequently develop focal segmental glomerular syndrome (FSGS) and renal failure, which are usually detected in the late stages and lead to the patient’s death. This process may be linked to the role of MAFB in controlling the differentiation and development of podocyte foot processes, as well as the survival of renal tubules (19). As proof of the importance of *MAFB’s* role, mutations in its leucine zipper domain also lead to FSGS with Duane retraction syndrome (22). A recent study showed that FSGS patients and those with renal diabetic status present low levels of MafB protein in podocyte cells (23). However, the relationship between *MAFB* variants and nephropathy in MCTO patients is unknown.

To date, little is known about the pathogenicity of MCTO disease, particularly the mechanism involved in bone osteolysis and renal failure, which is a consequence of this genetic disorder in most patients. In this study, we investigated the genetic etiology and clinical particularities of MCTO syndrome in three siblings. We also explored the immune status and expression of candidate genes implicated in the processes of osteoclastogenesis, inflammation, and oxidative stress to better understand the pathophysiological mechanism of this disease. The ultimate goal is to establish a genetic diagnosis, facilitate the management and follow-up of this disorder, and explore the potential contributions of genetic and immunological alterations to the pathophysiology of MCTO.

## 2 Materials and methods

### 2.1 Clinical data collection

This study was conducted in accordance with the Declaration of Helsinki principles and approved by the Bio-medical Ethics Committee of Pasteur Institute of Tunisia (Ethical Approval Reference 2021/10/E/V1). After obtaining written informed consent from the patients and their tutors, blood samples were collected in the pediatric orthopedic department of the Hospital Bechir Hamza. Three siblings were enrolled in the study and underwent routine general examinations for years. Clinical and genealogical data were collected under the supervision of the referral clinician. Radiography, biochemistry, and histological tests were performed for all patients suspected of MCTO syndrome.

### 2.2 Genetic investigation and in silico analysis for variant classification

Blood samples were collected from probands and their mother. DNA extraction was performed using the FlexiGene DNA Kit, according to the manufacturer’s instructions. The amplification of the *MAFB* gene was performed using the primers designed by Zankl et al. (2). Then the genetic investigation was performed using the Sanger sequencing technique.

Variant pathogenicity assessment was performed according to the American College of Medical Genetics (ACMG) guidelines. We confirmed the rarity of this variant through GenomAD and the 1000 Genome project, and we used 13 different prediction tools to predict the pathogenicity of the detected variant (**Supplementary Table 1**).

We also investigated whether this variant affects the phosphorylation process of the MafB protein using NetPhos prediction tool 3.1, and its interaction with 17 different kinases.

### 2.3 Candidate gene expression analysis

Total RNA was isolated from peripheral blood mononuclear cells (PBMC) from all patients and five healthy age-matched donors using Trizol reagent (No.: BCBL7327V, Sigma Aldrich, Darmstadt, Germany). cDNA was obtained by reverse transcription using reverse transcriptase (Superscript II, #18064014, Invitrogen), according to the manufacturer’s instructions.

We tested the expression of the different genes using the SYBR Green-Based qPCR. Primers were selected from the PrimerBank database (https://pga.mgh.harvard.edu/primerbank/) for the following candidate genes (*MAFB, IRF8, OPG, RANKL, TNFα, P65, P38, Il33, CD163, CD206, CD86, C3, IGF1, iNOS, ALOX12, PRDX3, FOXO3*). The samples were tested in duplicate by qPCR using LightCycler 480 SYBR Green I Master Mix (No: 04 707 516 001, Roche, Darmstadt, Germany), according to the manufacturer’s instructions. Q-PCR was performed using the LightCycler 480 System (Roche Diagnostics). Relative quantification Ct values were obtained from the threshold cycle number of a duplicate test for both, the target genes and housekeeping genes. The normalization was performed using the ΔCt method relative to two housekeeping genes (PPIA and RLP0), ensuring correction for sample-to-sample variation. The final result was presented by the log10 of the relative gene expression, with normalization to the mean of the healthy donor group, using the 2-ΔΔCT cycle threshold method for quantitation. Due to the limited cohort size, results are presented descriptively, without statistical testing. Given the rarity of MCTO samples, changes in gene expression were considered indicative of relative over- or under-expression if the log10 (fold change) was within this threshold (0.3 < log 10 (fold change) < -0.3).

### 2.4 Flow cytometry

Blood samples were collected from five healthy donors and from the three MCTO patients. PBMC were extracted using density gradient centrifugation on a Ficoll cushion. For each sample, 200,000 PBMCs were labeled using the following antibodies from BD biosciences firm: anti-CD14 APC-Cy7 (clone MΦ¨P9, # 557831), anti-CD16 FITC (clone 3G8, # 555406), anti-CD86 APC (clone 2331 (FUN-1), #555660), anti-CD4 PE-Cy5 (clone RPA-T4, #555348), anti-CD8 FITC (clone RPA-T8, #555366), anti-CD27 PE (clone M-T271, #555441), anti-CD28 APC-H7(clone CD28.2, #561388), anti-CD3 APC (clone HIT3a, #555342), anti-CD19 FITC (clone HIB19, #555412), anti-CD20 PE-Cy5 (clone 2H7, #555624), anti-CD56 PE-Cy7 (clone B159, # 557747).

**Figure 1:**
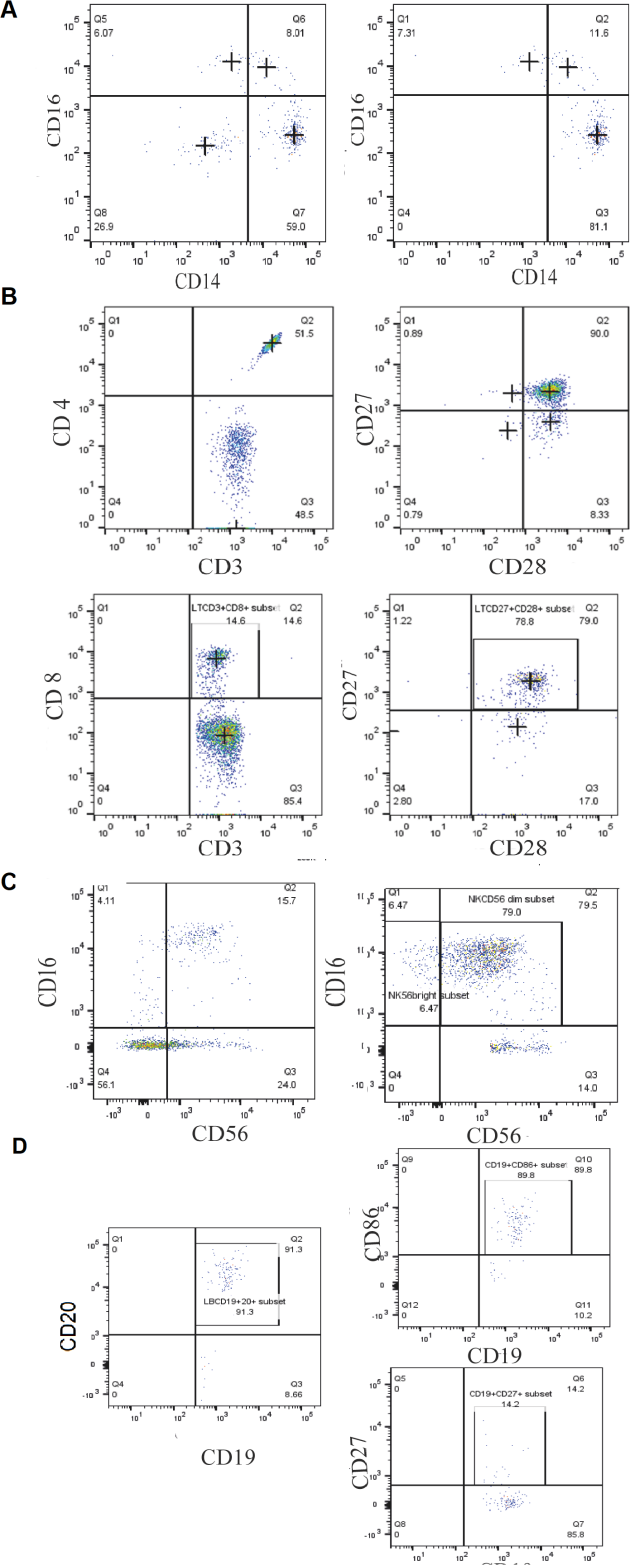
Gating strategy of different blood cell populations in MCTO patients, based on the differential expression of the different surface markers. **(A)** Monocyte subsets (CD14, CD16) **(B)** the T lymphocytes subtypes (CD4, CD8, CD27, CD28); **(C)** NK cell subtypes (CD16, CD56) and; **(D)** B lymphocytes subtypes (CD19, CD20, CD27, CD86).

Plot acquisition was performed using a flow cytometer FACSCanto™ II (BD Biosciences) for at least 10.000 events per sample. Experiments were performed in triplicate to ensure reproducibility. Data were analyzed using FlowJo v10 software. The gating strategy was performed according to previously published papers (24, 25). Parent populations were sequentially gated as illustrated in **Figure 1**. For each patient and control, gating was performed consistently by the same operator to ensure reproducibility. Subset frequencies are reported as percentages of the parent population, and due to the limited cohort size, the results were presented descriptively without formal statistical testing.

### 2.5 Quantification of cytokines in patients’ serum

The serum was collected from the blood samples and diluted to the appropriate concentration. Twelve cytokines/chemokines (IL1α, IL1β, IL2, IL4, IL6, IL8, IL10, IL12, IL13, IL17α) and granulocyte macrophage-colony stimulating factor (GM-CSF) were measured simultaneously using the Enzyme-linked immunosorbent assays (ELISA) technique, which were performed through Multi-Analyte ELISArray Kits (Cat no. MEH006A, Qiagen, US), following the manufacturer’s instructions. Given the small number of patients and healthy donors, these data are presented descriptively. We set a threshold change for serum level changes and considered cytokine secretion levels above three times the fold of donors as a differentially high serum level.

## 3 Results

### 3.1 Clinical data of studied patients

**Figure 2:**
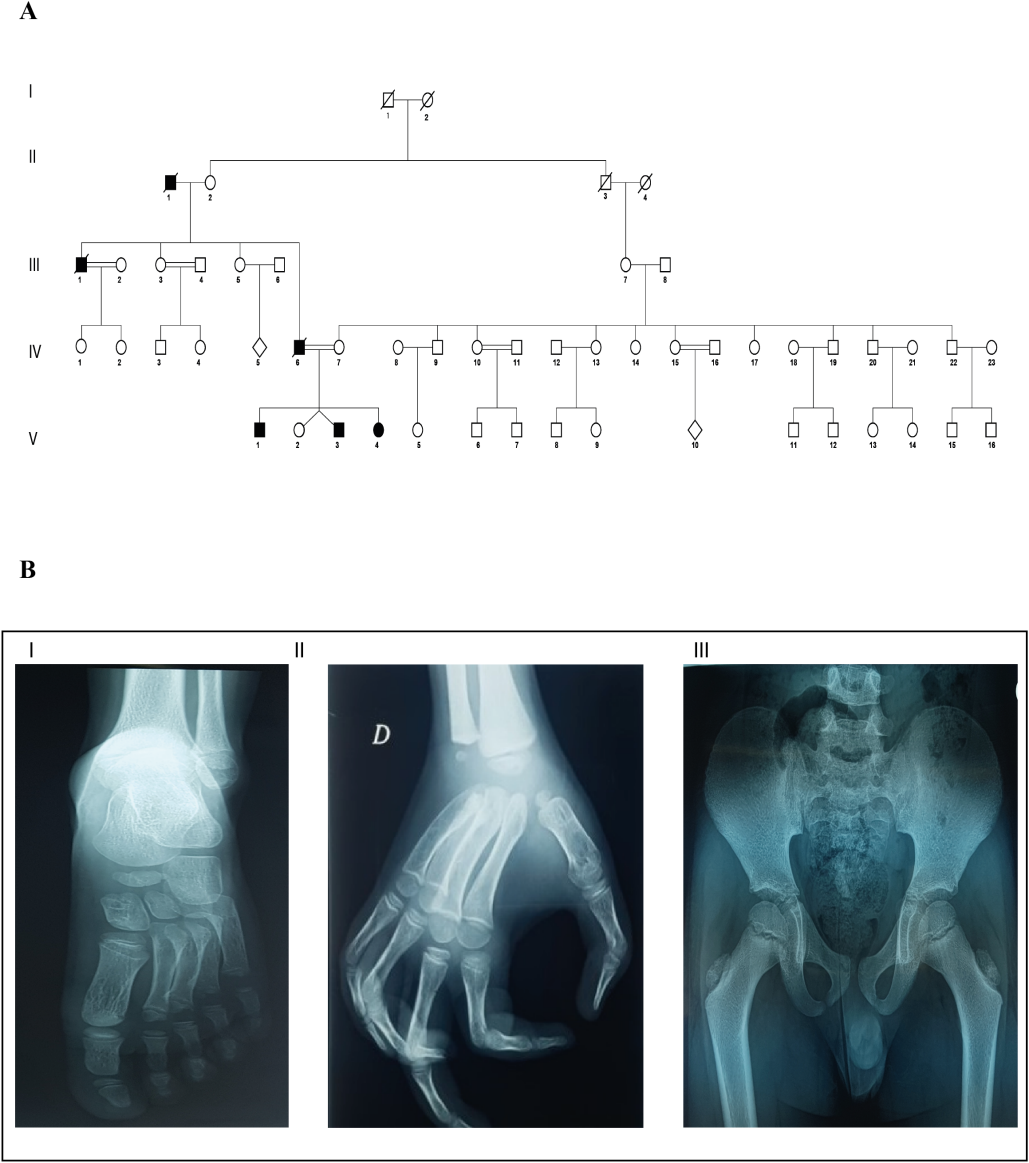
Clinical and genealogical characterization of MCTO in the affected family **(A)** Pedigree describing affected MCTO family member: Filled symbols represent affected individuals, open symbols represent unaffected individuals; **(B)** X-ray view of bone osteolysis of the affected MCTO family members: I: Erosion of the tarsal bone; II: Loss of carpal bone; and III: coxa valga.

In this study, we report a consanguineous family from North Tunisia (Ortho13) with four children, three of whom were affected with MCTO syndrome, which appears to have been inherited from their father and grandfather (**Figure 2A**). This interpretation is based on the clinical phenotypes acquired during the genetic questionnaire on family history. The three patients were coded as Ortho13EA1, Ortho13EA2, and Ortho13EA3.

All patients presented facial dysmorphism, bone osteolysis, joint pain, swelling, and loss of motion (**Table 1**). Furthermore, the father and grandfather died following kidney failure after ineffective hemodialysis.

**Table 1:** Clinical, radiological, biochemical, and histological features of the three patients with MCTO disease.

Patientcode clinical signs	Ortho13EA1	Ortho13EA2	Ortho13EA3
Age	17	12	9
Sex	M	F	M
Age of first diagnosis	8 months	1 year old	5 years old
Carpal and tarsal osteolysis	Carpal and tarsal bones	Carpal bones	Carpal bones of the right wrist
Joint pain	+	+	+
Swelling and loss of motion	+	+	+
Inflammatory Markers (CRP, ESR, rheumatoid factor, autoantibodies)	Normal	Normal	Normal
Facial dysmorphism	Triangular face and hypotelorism	Triangular face and hypotelorism	Triangular face and hypotelorism
Nephropathy	+	–	–
Age at first kidney disease manifestation	17 years old	–	–
Proteinuria	Normal	Normal	Normal
Renal Histology	Focal segmental glomerulosclerosis	Normal manifestation	Normal manifestation
Other manifestations	Cavovarus Coxa valga	Lower limb length discrepancy	Intellectual disabilities
Treatments	- 2 Surgeries to correct the foot deformities - Rehabilitation exercises	- Orthopedic insole -Rehabilitation exercises	- Rehabilitation exercises

F, female; M, Male; +/- presence or absence.

#### 3.1.1 Patient ortho13EA1

Ortho13EA1 was diagnosed at the age of 8 months because of joint deformities. These deformities were associated with remarkable carpal and tarsal dissolution observed on radiological examination, in addition to the presence of a coxa valga phenotype. Furthermore, the patient presented cavovarus foot deformities for which two surgical treatments were performed, followed by postoperative administration of Ibuprofen for pain management. However, abnormal gait was maintained even after correction. This patient presented with progression in clinical features with age, especially regarding bone vanishing (**Figure 2B**). Moreover, at the age of 17 years, a renal biopsy revealed early onset of FSGS.

#### 3.1.2 Patient Ortho13EA2

Ortho13EA2 was suspected to be affected with MCTO syndrome since the age of 1 year, because of the presence of carpal bone osteolysis and a deformity of the phalanges, which was managed using braces to support proper alignment and function. The patient also suffered from a lower limb-length discrepancy.

#### 3.1.3 Patient Ortho13EA3

Ortho13EA3 presented a later onset of the disorder (5 years-old). He presented osteolysis in the carpal bones of the right wrist. In contrast to the other siblings, this patient was also diagnosed with an intellectual disability.

### 3.2 Genetics results

**Figure 3:**
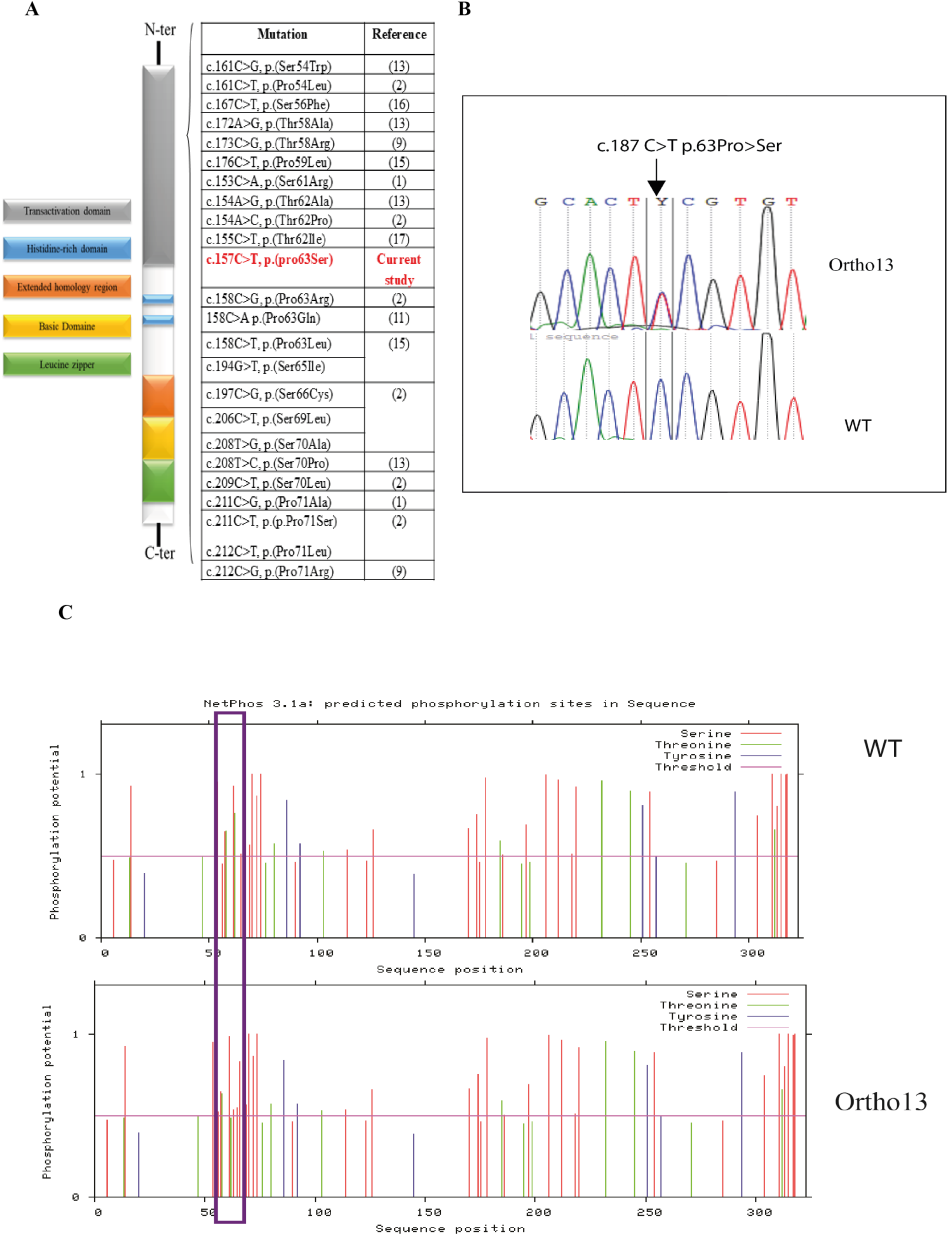
Genetic alterations of *MAFB* associated with MCTO syndrome. **(A)** Schematic representation of the variant detected in MAFB gene in our patients and the list of other variants associated with MCTO syndrome; **(B)** Electropherograms of the variant c.187C>T in the MAFB gene: Ortho13: Presents the electropherogram of MCTO patients and WT presents the electropherogram of the healthy sibling; **(C)** Phosphorylation site prediction of the MafB protein in the wild-type protein (WT) and in the mutated form (ortho13) using NetPhos 3.1 software: Prediction result differences are delimited in the purple rectangle.

Direct Sanger sequencing of the *MAFB* gene (NM_005461.5) revealed a novel heterozygous variant (c.187C>T; p.(63Pro>Ser)) was detected in all three patients. The mother and healthy sibling did not carry this variant (**Figure 3B**). This *MAFB* variant has not been previously reported (**Figure 3A**); however, it was predicted to be pathogenic using multiple prediction tools (**Supplementary Table 1**) and we classified it as pathogenic according to the ACMG criteria, with supporting evidence codes PM1, PM2, PM5, PP1, PP3, and PP4.

### 3.3 In silico study of the effect of the variant on MafB protein phosphorylation

As MafB protein activity is controlled by the phosphorylation of different amino acids, we studied the effect of this variant on its phosphorylation using the NetPhos prediction tool 3.1. We found a loss of the phosphorylation site at the position p.62Thr, which was phosphorylated by GSK3, Unsp, P38MAPK, and cdk5, leading to the creation of a new phosphorylation site at the position p.63Ser due to the new variant, which may be phosphorylated by cdc2 (**Figure 3C**).

### 3.4 Candidate genes’ expression

In order to decipher the molecular pathophysiology of MCTO disease, we investigated three different pathways implicated in osteoclastogenesis in a limited set of genes: *MAFB*, RANKL-induced osteoclastogenesis-related pathway, oxidative stress, and inflammation-related genes.

We observed variable *MAFB* expression levels in the different siblings. For patient Ortho13EA1, an under-expression of this gene was detected (log10 fold change = -1,32 ± 0.2); however, an overexpression was observed for patients Ortho13EA2 and Ortho13EA3 (log10 fold change = 1.49 ± 0.2 and 1.65 ± 0.06, respectively) compared to healthy donors.

*IRF8* and *OPG*, inhibitors of osteoclastogenesis, were similarly underexpressed in the three patients (log10 fold change = -1,92 ± 0,37 and -1,38 ± 0,19), whereas *RANKL* and *TNFα*, which are pivotal molecules for osteoclastogenesis, were overexpressed (log10 fold change = 1,029 ± 0,09 and 1,16 ± 0,15). As for inflammation-related genes, the sets of *P65*, *P38, and IL33*, which mediate signaling cascades essential for osteoclast differentiation and regulate bone remodeling, were overexpressed compared to healthy donors, with a log10 fold change value of (0,42671 ± 0,04; 0,91± 0,01 and 1,75 ± 0,46), respectively.

In addition, given the implication of the oxidative stress pathway in osteoclastogenesis, we studied the expression of oxidative and antioxidative genes. An overexpression of oxidative genes was noted for *iNOS* (log10 fold change value = 2,23 ± 0,25), *ALOX12* (fold change value = 1,95 ± 0,21 and *FOXO3* (log10 fold change value = 1,49 ± 0,37), while an underexpression of the anti-oxidative gene *PRDX3* was found (log10 fold change value = -1,96 ± 0,21).

**Figure 4:**
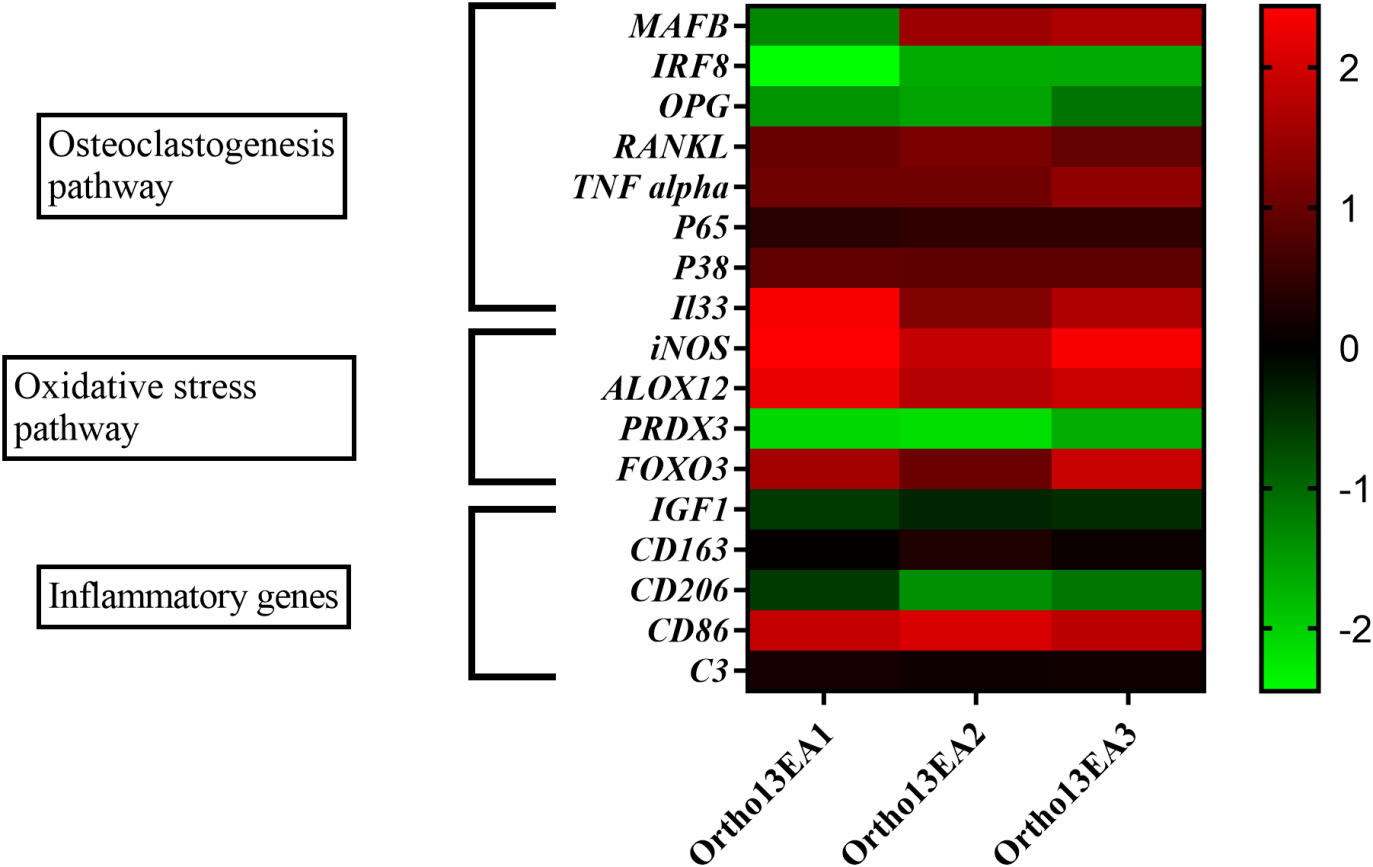
Heatmap of 17 genes expressed in log10 fold change of MCTO patients. Presentation of 3 major pathways showing variability between the patients.

We also examined the expression of inflammatory genes related to macrophage polarization, given the role of *MAFB* in this process. An over-expression of the pro-inflammatory gene that codes for *CD86* was observed (log10 fold change = 1,9 ± 0,11), while anti-inflammatory genes *CD206* and *IGF-1* were under-expressed compared to healthy donors with log10 fold change values of -1,02 ± 0,3 and -0,44 ± 0,08, respectively. However, a normal expression was observed for *C3* and *CD163* genes with a log10 fold change value of 0,17± 0,05 and 0,13± 0,1 (**Figure 4**).

### 3.5 Alteration of immune cell phenotype due to *MAFB* variation

**Figure 5:**
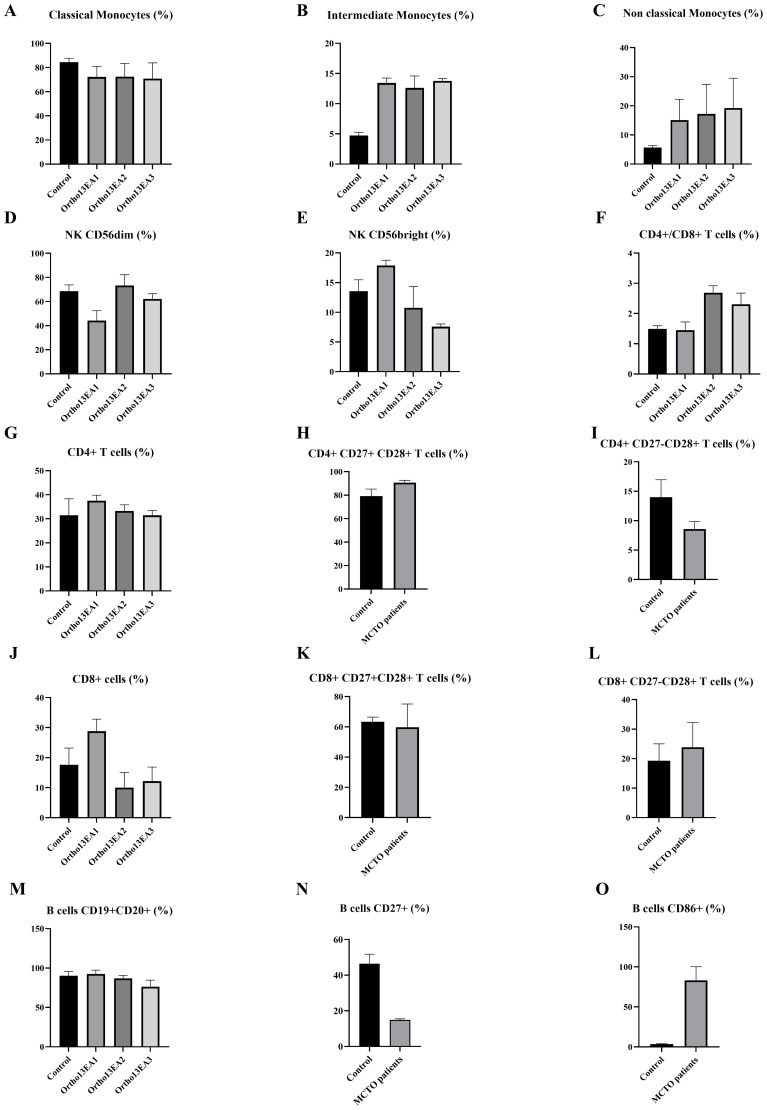
Characterization of immune cell phenotypes in MCTO patients compared to healthy donors. **(A)** Percentage of CD14++CD16- Classical monocytes; **(B)** Percentage of CD14++CD16+ intermediate monocytes; **(C)** Percentage of CD14+CD16++ non-classical monocytes; **(D)** Percentage of NKCD56^dim^ cells; **(E)** Percentage of NK CD56^bright^ cells; **(F)** Ratio of T CD4+/CD8+ cells; **(G)** Percentage of T CD4+ cells; **(H)** Percentage of early differentiated T CD4+CD27+CD28+ cells; **(I)** Percentage of intermediate-differentiated T CD4+CD27−CD28+ cells; **(J)** Percentage of T CD8+ cells; **(K)** Percentage of early differentiated T CD48+CD27+CD28+ cells; **(L)** Percentage of intermediate-differentiated T CD8+CD27−CD28+ cells; **(M)** Percentage of B cells; **(N)** Percentage of B CD27+ B cells; **(O)** Percentage of B CD86+ cells.

Flow cytometry analysis was performed to assess the percentages of monocytes, T and B lymphocytes, as well as NK cells in the MCTO patients, performed in triplicate for each patient and compared to five age-matched healthy donors (**Figure 5**).

For innate immunity, we investigated the different monocyte subsets and noted lower frequencies of classical monocytes (CD14++CD16-) with a percentage of 77.81% ± 2.87 compared to 84,45% ± 2,96 in healthy donors. However, patients exhibited higher frequencies of intermediate monocytes (CD14++CD16+) and non-classical monocytes (CD14+CD16++) with a percentage of 13,26% ± 2,49 and 12,143% ± 1.35, compared to healthy donors 4.73% ± 1,257 and 5,7% ± 0,66, respectively (**Figures 5A–C**). As for NK cells, an increased percentage of NK CD56^bright^, particularly in the patient Ortho13EA1 who presented the most severe clinical manifestations, was noted, with a percentage of 22,15% ± 7,44 compared to healthy donors 13,56% ± 1,71, and a lower frequency of NK CD56dim 48,8% ± 1,9, compared to healthy donors 68,5% ± 4,72 (**Figures 5D, E**). The same patient, Ortho13EA1, showed a tendency toward an increased percentage of CD8+ T cells of 31,1% ± 0,1 compared to healthy donors 17,61% ± 5,27. The global percentage of B cells in the three patients was comparable to that in healthy donors. However, CD86+ B cells appeared to be higher in MCTO patients, with a percentage of 83,16% ± 13,91 compared to healthy donors 3,61% ± 0,36. In contrast, CD27+ B cells seemed to decrease in our patients, with a percentage of 14,93% ± 0,49 compared to healthy donors 46,38% ± 4,80 (**Figures 5M–O**).

### 3.6 Alteration in the expression of IL8 cytokine

**Figure 6:**
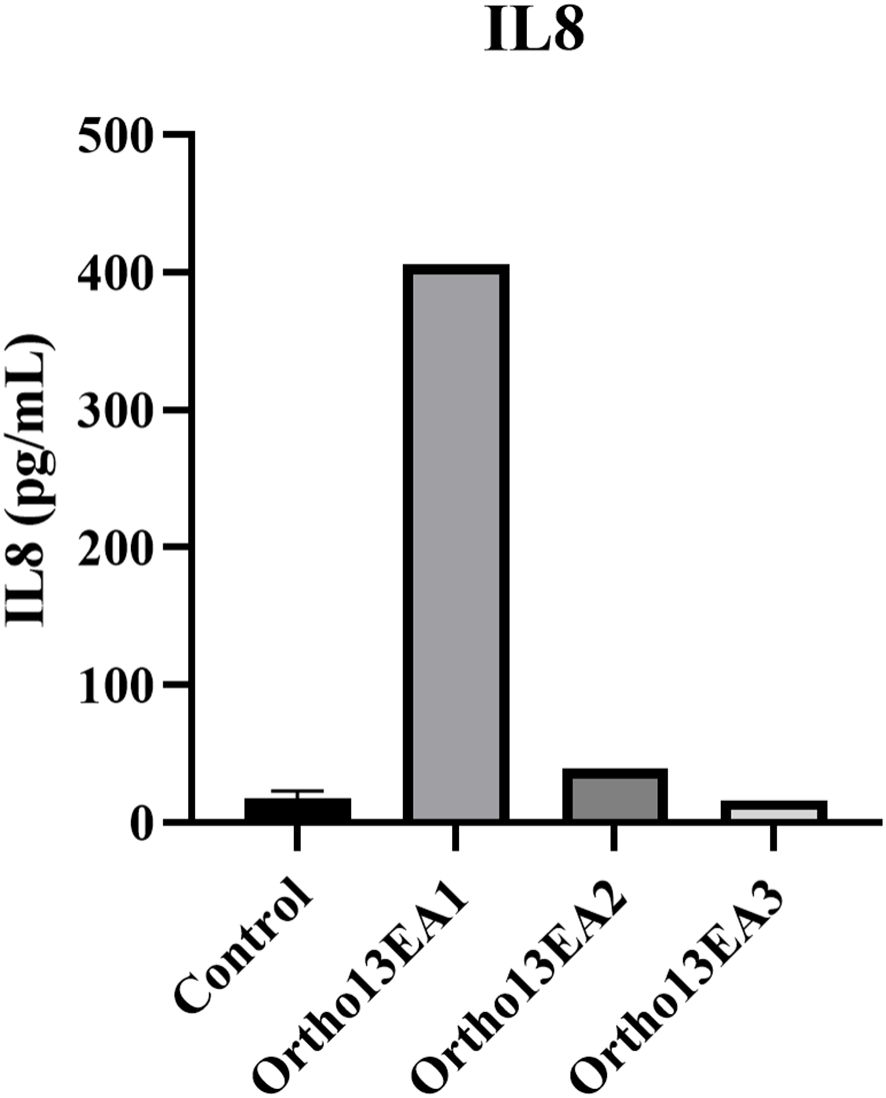
Concentration of IL8 (pg/mL) in the serum of MCTO patients compared to healthy donors.

Twelve cytokines (pro- and anti-inflammatory cytokines) were measured using a multi-analyte ELISA-Array kit in MCTO patients and healthy donors. Interestingly, only an increased level of IL8 was noted in the patient ortho13EA1 (406,205 pg/mL) compared to healthy donors (17,59 pg/mL ±4,71) (**Figure 6**).

## 4 Discussion

### 4.1 Clinical and genetic investigations

Multicentric carpotarsal osteolysis syndrome is a rare genetic skeletal dysplasia that begins in early childhood and mimics JIA. It is characterized by progressive bone resorption, especially in the carpal and tarsal bones, and can affect other joints. In most cases, this disease is followed by nephropathy and renal failure (2). However, conventional JIA treatments are not recommended for patients at risk of developing nephropathy.

In this study, we reported the first cases of MCTO syndrome Tunisian patients. According to the clinical information obtained following the genetic inquiry, we hypothesized that this disease was inherited from the paternal genealogy. All our patients presented with the clinical features of MCTO disease, which is characterized by bone osteolysis, joint pain, and loss of mobility (1). These skeletal manifestations show notable overlap with the clinical features of JIA, particularly with joint pain and deformity, swelling, and limited range of motion, highlighting the risk of misdiagnosis and delayed diagnosis of MCTO, which was estimated at 3.82 years (ranging from 0 to 35 years) because of its rarity (18). However, these manifestations can lead to secondary injuries, progressive disability, and functional limitations (26).

In our clinical approach, after confirming the disease, patients were educated on personalized rehabilitation exercises to perform it independently at home. The goal of this approach is to enable patients to manage disease progression and maintain joint mobility. Similar strategies for home-based exercises have been successfully implemented in the management of JIA, where programs have been shown to significantly improve pain, physical function, and quality of life in affected children (27). However, rehabilitation remains largely overlooked in the literature. A recent study highlighted the importance of rehabilitation in MCTO management by introducing the “Function Profile” framework, emphasizing the need for systematic evaluation and customized interventions (26).

In addition to skeletal manifestations, patients with MCTO may present with craniofacial abnormalities, such as triangular faces, maxillary hypoplasia, and exophthalmos or hypotelorism (5, 8). Triangular faces and hypotelorism were observed in our patients, consistent with previous studies. Interestingly, intrafamilial variability was observed, which could be explained by age differences or the involvement of other epigenetic factors. Similar cases have been reported in the literature for patients carrying the same *MAFB* variants, presenting clinical heterogeneity involving skeletal features, renal phenotypes, and neurodevelopmental manifestations (8, 15, 17). To date, there is no genotype–phenotype correlation (18), and this intra- and inter-familial heterogeneity could be associated with other factors beyond the primary mutation in *MAFB*, such as epigenetic regulation. In fact, DNA methylation and microRNA-mediated regulation play important roles in modulating *MAFB* expression and may be involved in clinical variability. Interestingly, a recent study demonstrated that MAFB can work with the glucocorticoid receptor to remodel the epigenome signature in tolerogenic dendritic cells, influencing the DNA methylation patterns of other genes to modify their transcription (28). Furthermore, the implications of gene modifiers have been widely investigated in rare Mendelian disorders for their potential to influence phenotypic variability and disease severity (29). However, no study has investigated these modifications to explain the phenotypic variability of MCTO disease. Future research on the epigenetic landscape of MCTO is required to understand this variability.

Ortho13EA1, the oldest sibling, exhibited the most severe manifestations among the siblings. This patient had a foot deformity that prevented him from walking properly and required surgical intervention. Cavovarus foot has been previously described in one patient with MCTO, but it was not associated with severe manifestations (17). In addition, coxa valga was observed, especially in the right femoral neck, which has not been previously described in MCTO patients, and contributed to the limitation of abduction and internal rotation of the hip. For Ortho13EA2, asymmetry of the lower limbs was observed, causing postural imbalance. This clinical feature has not been previously described in patients with MCTO. For Ortho13EA3, bone erosion was observed only in the right wrist. This has been described in two patients without progression to other joints (5). It is interesting to note that the expression of the disease was late compared to his siblings and manifested only following the death of his father. In fact, it has been described in the literature that psychological stimuli related to stress and emotional trauma can promote neuroendocrine dysfunction and inflammation, contributing to the pathogenesis and expression of diseases such as rheumatoid arthritis, multiple sclerosis, and systemic lupus erythematosus (30). Furthermore, Ortho13EA3 was diagnosed with intellectual and learning disabilities, which are rare clinical signs of MCTO disease, observed in two patients in the literature (5).

Regarding the genetic investigation of this disorder, heterozygous missense variants in the N-terminal region of *MAFB* have been associated with MCTO. Interestingly, all variants are located in a conserved region of the protein (54–71), which encodes a transactivation domain clustered at the phosphorylation site. In detail, this conserved region plays an important role in all large MAF family proteins. Its phosphorylation results in both increased transactivating function and decreased protein stability (18). Variants in this region could, therefore, affect protein stability and activity. To date, 23 missense variants have been associated with MCTO disease (**Figure 3A**) (1, 9, 11, 13, 16).

In this study, we describe a novel missense variant detected in this conserved region, c.187C>T; p.(63Pro>Ser) associated with MCTO in three patients. In fact, a variant in the same amino acid c.188C>G; p.(Pro63Arg) has been previously reported to be associated with MCTO syndrome (2). Moreover, it was shown that this region of the MafB protein is phosphorylated via the action of GSK3 kinase at the following N-terminal residues (Ser66, Thr62, Thr58, and Ser54), which enhances their transactivation activity via recruitment and binding with p300-CBP co-activator proteins (18). GSK3 specifically phosphorylates Ser/Thr amino acids, suggesting that the transversion of proline to serine in our patients supports the hypothesis of the creation of a novel phosphorylation site. In order to confirm this hypothesis *in silico*, we used the NetPhos server, a neural network-based method for predicting potential phosphorylation sites at serine, threonine, or tyrosine residues in protein sequences. The overall results indicate that the variant detected in our patients, even though it doesn’t affect a phosphorylation site, could modify the protein conformation, which affects the phosphorylation of the Thr62 residue, causing a loss of interaction between GSK3 and the Thr62 phosphorylation site, which could decrease the transactivation activity of the MafB protein. Moreover, the replacement of Pro63 with Ser residue creates a new phosphorylation site that is predicted to interact with cdc2 protein kinase, which could affect the stability and activity of the protein. In addition, different prediction tools have predicted that this variant may decrease protein stability. While our genetic investigations support the pathogenic effect of the *MAFB* variant, further functional studies are required to confirm its mechanistic impact.

### 4.2 Molecular and cellular investigations

Multicentric carpotarsal osteolysis syndrome is often misdiagnosed as idiopathic inflammatory arthritis in children (18). However, conventional treatments for Juvenile Idiopathic Arthritis (JIA) are ineffective and harmful to patients at risk of developing nephropathy. Furthermore, because of the delayed onset of nephropathy and primary onset of bone osteolysis, nephropathy diagnosis is delayed, resulting in later interventions in the advanced stages of the disease.

Given the rarity of MCTO disease, little is known about the pathophysiology of bone osteolysis and the causes behind the occurrence of nephropathy. In this study, we deeply investigated the molecular and cellular profiles of patients with MCTO disease, which could help in better understanding disease manifestations, particularly bone osteolysis and nephropathy.

#### 4.2.1 Gene expression profile related to osteogenesis, inflammation, and oxidative stress in MCTO patients

Variable expression of the candidate genes was observed in the affected sibling. First, we studied the gene expression involved in the ossification mechanism. In fact, the balance between bone matrix formation and bone resorption is crucial for healthy bone metabolism, and these processes are tightly regulated by various hormones and cytokines. Dysregulation of these processes leads to different bone disorders, such as RA and osteoporosis. Thus, we studied the expression of *MAFB*. Interestingly, *MAFB* expression varied among the patients. Under-expression was detected in the older brother (Ortho13EA1), whereas overexpression was detected in the other two siblings. This variability could be explained by the involvement of epigenetic factors, as in patients with rheumatoid arthritis (28). Interestingly, variants in the N-terminal region of *MAFB* leading to MCTO overlap with a CpG island, suggesting that DNA methylation may modulate its expression or transcriptional activity (31). In addition, *MAFB* expression is modulated by miRNAs, such as miR-155 and miR-148a, which directly target *MAFB* mRNA by binding to the 3’UTR and repressing MafB protein expression in macrophages, favoring a pro-inflammatory state and promoting osteoclast differentiation (19, 32). These epigenetic modifications may be implicated in the variable expressivity of *MAFB* and the low expression observed in the Ortho13EA1 patient.

Besides, *MAFB* expression and activity can be inhibited by miR-320a, which was proven to lead to podocyte injury in a diabetic mouse model, and restoring *Mafb* attenuates this injury (33). Moreover, underexpression of *MAFB* was noted in patients with Focal Segmental Glomerulosclerosis (FSGS) with Duane Retraction Syndrome, who presented with *MAFB* variants, and its underexpression was associated with nephrological manifestations (22). In addition, recent studies have shown downregulation of *MAFB* in patients with FSGS, chronic kidney disease (CKD), and diabetic kidney disease (23). Consequently, we suspected renal dysfunction in ortho13EA1. We referred our patients to a nephrologist who diagnosed early renal dysfunction and focal glomerulosclerosis lesions in patient Ortho13EA1, contributing to early detection. Kidney defects in MCTO patients are a common feature. Renal impairment ranges from elevated proteinuria to renal failure, requiring hemodialysis and renal transplantation. In the literature, 43 MCTO patients (60%) presented with renal dysfunction (1, 3, 4, 7–10, 13, 14). In our case, renal failure was the cause of death in both the father and grandfather. Therefore, no genotype-phenotype correlation has been established to date (5). In our specific case, we suspected that the onset of nephropathy in ortho13EA1 was triggered by the use of ibuprofen, a non-steroidal anti-inflammatory drug, administered to the patient after foot surgery to manage pain. The use of non-steroidal anti-inflammatory drugs (NSAIDs) is recommended for patients with JIA; however, it is harmful to patients with MCTO because of the risk of developing nephropathy (18). Although no direct link between NSAIDs and *MAFB* expression has been established, experimental models have indicated that COX/prostaglandin signaling modulates MAFB activity in macrophages during the recovery phase of acute kidney injury (34). However, studies have demonstrated that NSAIDs inhibit the COX pathway (35). Future studies should investigate the regulation of *MAFB* expression in response to environmental factors, such as NSAID use, and the effect of epigenetic modifications and their implications for variable clinical features in MCTO patients.

The progressive osteolysis in our patients could be caused by impaired osteoclastogenesis and thereby an unbalanced formation of osteoblasts and osteoclasts leading to bone erosion, as mentioned in recent studies on MCTO disease (12, 31). To investigate this hypothesis, we studied the expression of genes related to the ossification process. In detail, we studied the expression of *RANKL, TNFα* and their signaling pathway. In fact, RANKL and TNFα are both implicating in the osteoclast differentiation from monocytes/macrophages via activating the nuclear factor-κB (NF-κB) and the mitogen-activated protein kinases (MAPKS), which inhibit MafB activity and activate other transcription factors leading to osteoclastogenesis (36). In our study, *RANKL* and *TNFα* were overexpressed in all the patients compared to the healthy donors, which is in favor of the hypothesis of excessive osteoclast formation. Similarly, studies of JIA showed high expression of RANKL and TNFα, where they contribute to osteoclast differentiation and activity, thus promoting bone resorption and osteolysis (37). Therefore, we hypothesize that even in the case of the overexpression of *MAFB*, which could inhibit RANKL activity, the osteoclastogenesis could be induced by TNFα in MCTO patients. Moreover, we investigated the expression of *P65* and *P38*, which are major subunits of the NF-κB and MAPK proteins. An overexpression of *P65* and *p38* was noted in all our patients compared to healthy donors, which is in line with our hypothesis. Alteration of the NF-κB signaling pathway and the high expression of *P38* were detected in the joints of RA patients. Several treatments for RA were based on inhibiting the NF-κB pathway, which can suppress bone erosion (38, 39). These alterations could therefore be associated with MCTO disease development. Furthermore, we assessed the expression of the negative regulator genes of osteoclastogenesis, such as the osteoprotegrin (OPG) and Interferon regulatory factor-8 (IRF-8) genes. An underexpression of these genes was detected in MCTO patients. In fact, IRF-8 deficiency promotes TNFα-induced osteoclastogenesis (40). In addition, the underexpression of the OPG/RANKL ratio was associated with RA and JIA pathogenesis (41). Similarly, our results showed that the ratio OPG/RANKL is lower than ≤0.5.

Cytokines play an important role in osteoclastogenesis, especially IL33, a member of the IL1 family, which induces the production of pro-inflammatory mediators such as TNFα and IL1-β involved in inflammation-induced bone destruction. A high level of the IL33 cytokine was detected in the serum of JIA, RA, and spondylarthritis patients. A correlation between IL33 in the serum and the disease activity was described (42). In our study, we detected a high expression of the *IL33* gene in the MCTO patients. Furthermore, a previous study suggests an increased expression of *IL33* in patients with chronic renal disease (43). In our case, the highest expression level was detected in Ortho13EA1, which also exhibited an early onset of nephropathy.

During osteoclastogenesis, the implication of the ROS component and its interaction with P38 and JNK was reported. Oxidative stress is an imbalance between ROS production and/or decreased antioxidant defense activity. Many bone diseases are related to oxidative stress, especially RA (44). For that, we studied the expression of *iNOS*, which codes for one of the isoforms of nitric oxide synthase. Increased *iNOS* expression was observed under oxidative stress, favoring excessive osteoclast differentiation. Their overproduction in blood and synovial fluid has been associated with RA pathogenesis (45). Similarly, in our study, we observed an overexpression of *iNOS* in MCTO patients. Furthermore, we studied the expression of *ALOX12*, which codes for one of the oxidative stress enzymes producing the lipid peroxides. The overactivity of this gene was associated with the development of osteoporosis. The expression of *ALOX12* was increased in our patients. Moreover, we observed a low expression of *PRDX3*, which codes for a mitochondrial protein with antioxidant function. Similar results were detected in patients with RA (46).

Several defense mechanisms, such as autophagy and apoptosis are induced in response to oxidative stress in bone cells. Many pathways, such as ROS/FOXO3, ROS/AMPK, and ROS/JNK/c-Jun are also implicated. FOXO3a functions as a trigger for apoptosis and autophagy through the expression of genes necessary for cell death. The expression of the *FOXO3a* gene in our patients was higher than in the healthy donor group. Similar results were observed in RA patients (47). However, the mechanism of action of FOXO3a in MCTO and RA patients remains unclear. On the other hand, FOXO3 is negatively regulated by insulin growth factors. Stress stimuli by depletion of growth factor induce the activation of FOXOs, including FOXO3a. Moreover, IGF-1 also plays an important role in bone homeostasis and promotes osteogenesis in both endocrine and paracrine manners. An under-expression of this gene was detected in our patients. Similar results were detected in MCTO patients (6). Therefore, this gene plays an important role in inflammatory bone diseases. In these pathologies, inhibition of *IGF-1* expression by TNFα leads to bone loss (48).

Given the role of *MAFB* in the differentiation of monocytes to macrophages with an anti-inflammatory phenotype, we investigated the expression of *CD86* and CD206 cell markers. A high expression of the *CD86* gene vs a low expression of the *CD206* gene was noted in our patients compared to donors. In fact, pro-inflammatory monocytes expressing CD86 play an important role as osteoclast precursors (40). Furthermore, monocytes in RA patients showed defective polarization of monocytes toward M2-like macrophages with decreased expression of the CD206 surface marker, which was negatively correlated with the disease activity (49), and similar to our results. The polarization of monocytes toward the M1-like phenotype presented a high level of inducible nitric oxide synthase (iNOS) and pro-inflammatory cytokines (50), showing a similar pattern to what we observed in our findings.

Overall, our results suggest overlapping inflammatory and osteoclastogenesis-related pathway signatures with RA/JIA, which could be associated with bone osteolysis in MCTO. Moreover, the reduced *MAFB* expression observed in one patient may indicate a potential role in the onset of nephropathy. These findings require further confirmation in larger cohorts, as well as validation through functional studies.

#### 4.2.2 Immune cell phenotyping in MCTO patients

Inflammation has been extensively studied in RA patients, particularly the role of adaptive and innate immune cells, which exacerbate osteoclast activity and play a major role in bone osteolysis (20). However, despite the similarities between RA and MCTO, the immune cell status in MCTO remains largely unexplored. Furthermore, in the literature, some MCTO patients presented inflammation in the joints using musculoskeletal ultrasound and MRI, as well as the use of anti-inflammatory treatments, showing relief from joint pain (18), which is in favor of the hypothesis of the installation of inflammation in these patients despite normal biological parameters. Therefore, we studied the immune cell phenotypes in our patients, which have never been studied in this disease.

We first studied the monocyte profile in MCTO patients. In fact, monocytes are classified into three subsets based on the expression of CD16 and CD14. Previous studies show that *MAFB* is highly expressed only in CD16+ monocytes and monocyte-derived macrophages, supporting a role for this transcription factor in the differentiation and function of these subsets (51). In this study, the immunophenotyping of the monocyte subsets in our patients showed a trend toward an increased level of intermediate and non-classical monocytes (CD16+). Similar observations were noted in arthritis patients, in which these monocyte populations were associated with joint destruction. This increased percentage of both intermediate and non-classical monocytes was also described in peripheral blood and synovial fluid of patients with JIA and RA diseases (52).

Although there is no direct evidence linking *MAFB* variants to monocyte polarization, functional studies support the role of *MAFB* in directing monocyte differentiation into macrophages (53) and in inhibiting their differentiation into osteoclasts. Furthermore, *in vivo*, zebrafish *mafbb* mutants, homologous to human *MAFB*, exhibit enhanced osteoclast differentiation and skeletal defects similar to MCTO, and show that some described N-terminal *MAFB* variants fail to rescue these phenotypes. These findings support the notion that these variants related to MCTO are loss-of-function variants contributing to bone osteolysis (54). While our findings are limited to monocyte phenotyping in MCTO patients, further functional assays in human-derived cells are required to clarify the impact of *MAFB* variants on monocyte polarization and the association between monocyte subsets and bone osteolysis.

Furthermore, we studied the implication of lymphocytes in the pathophysiology of MCTO. We observed a high percentage of CD8+ T cells in the patient Ortho13EA1, who presents a severe phenotype with the FSGS manifestation. Moreover, it has been demonstrated that CD8+ T cells play an important role in chronic kidney diseases such as lupus nephritis and glomerulonephritis via inducing podocyte cell death (55). Further studies on a larger cohort are needed to show if CD8+ T cells are associated with FSGS in MCTO.

Regarding B cells, we show that our patients displayed a decreased percentage of the memory B cells (CD19+CD20+CD27+) compared to healthy donors. Similar results with a low percentage of B cells expressing CD27+ were observed in RA patients, and it was negatively correlated with the disease activity. However, after effective therapy with anti-TNFα antibodies, the number of memory B cells was significantly increased (56). On the other hand, we observed a high percentage of B cells expressing CD86 cell markers in our MCTO patients. This subtype plays an important role in the activation of T cells via the binding of CD86 to CD28, which enhances the secretion of RANKL (21). Furthermore, RA patients present a high frequency of CD86 in B cells, which participate in bone inflammation. Hence, decreasing the CD86 in B cells provides a new therapeutic mechanism for RA patients (57). Further studies are needed in MCTO patients to confirm the implication of B cell subsets with bone osteolysis in MCTO patients.

Regarding NK cells, a high percentage of NKCD56^bright^ was found only in patient Ortho13EA1, who presents with early nephropathy onset. In fact, a high level of NKCD56^bright^ cells was associated with interstitial fibrosis in patients with chronic kidney disease (58). Moreover, a decreased percentage of NK-CD56^dim^ cells has been reported in patients with CKD (59). A similar pattern was noted in patient Ortho13EA1.

All together, we suggest that the implication of intermediate, non-classical monocytes and differentiated B cell subtypes may be implicated in bone osteolysis observed in MCTO, which was similar to the immune alterations associated with the pathophysiology of RA/JIA. Moreover, we identified an alteration in cytotoxic T cells and NKCD56^bright^ cells in one patient, which could also be a potential factor for nephropathy onset. However, further multicenter studies are needed to validate whether this immunological status is associated with MCTO.

#### 4.2.3 Cytokines alteration in MCTO patients

Multiple inflammatory cytokines are involved in the pathogenesis of bone osteolysis (40). Therefore, we measured 12 cytokines in the serum of patients with MCTO. No significant alteration in cytokine levels was detected in the patients compared to healthy donors, except for IL8, which was increased in the patient Ortho13EA1, who exhibited a severe phenotype associated with FSGS onset. In the literature, it has been reported that IL8 is increased in patients with CKD, and it is considered a biomarker for CKD in the pediatric population (60). We suggest that IL8 could play a pivotal role in the onset of renal pathology in our patient. However, confirmation in a larger cohort and functional studies are required.

Overall, the clinical presentation of our patients highlights the variability within the MCTO spectrum, with clinical features overlapping with those of JIA. The identification of novel variants expands the mutation spectrum. Descriptive analyses of gene and protein expression and cellular immunophenotyping have provided similar pathways to those observed in RA/JIA, which may help to explain the phenotypic similarity. Furthermore, the phenotypic and gene expression variability observed among siblings may reflect epigenetic and environmental modifiers. However, this study included a small cohort, reflecting both the ultra-rare nature of MCTO and the potential underdiagnosis of this disease. The recruitment of age-matched healthy pediatric donors was limited by ethical and consent considerations. These elements restrict the statistical power and generalizability of our findings. However, a similar study was conducted in patient (6). To the best of our knowledge, this is the first multidisciplinary study to combine clinical, genetic, bioinformatic, and immunological investigations of MCTO, which may help guide future research on this disease. Further studies in larger cohorts, supported by functional assays and epigenetic analyses, will be necessary to validate our hypotheses and elucidate the biological role of MAFB.

## 5 Conclusions

We report a novel pathogenic variant that affects MafB protein conformation and phosphorylation in MCTO disorder. We further explored the clinical particularities of this rare disorder and identified potential factors that may be associated with bone osteolysis and the onset of nephropathy. We showed that patients with MCTO seem to exhibit an inflammatory status that may be responsible for these manifestations. We suggest that patrolling intermediate and non-classical monocytes with altered gene expression of key genes, such as *TNFα*, *IL33*, and *iNOS*, may contribute to bone osteolysis. With regard to nephropathy, we recommend avoiding the use of non-steroidal treatments, as these impair the immune response and aggravate the disease. Our findings provide insights for a better understanding of MCTO disorder, which would improve monitoring and management.

## Data Availability

The original contributions presented in the study are included in the article/**Supplementary Material**. Further inquiries can be directed to the corresponding author.
