# Supplementary material for: Uncovering the molecular and immunological defects in multicentric carpotarsal osteolysis syndrome: identification of relevant biomarkers
Source: Front Immunol. 2025 Oct 29;16:1543385. doi: 10.3389/fimmu.2025.1543385 (PMC12605331; doi:10.3389/fimmu.2025.1543385)
Supplement: Supplementary file 1 [file Table1.docx]

Supplementary Material

# Supplementary Table

Table 1: In silico prediction of the pathogenicity of the c.187C>T; p.(Pro63>Ser) variant in the *MAFB* gene

| **Prediction tools** | **Score** | **Significance** |
| --- | --- | --- |
| Mutation taster | 0,999 > 0,5 | Disease causing |
| UMD Predictor | 91 | Pathogenic |
| Provean | -4,34 < -2,5 | Deleterious |
| SIFT | 0,019 < 0,05 | Damaging |
| Mupro | -0,68 < 0  G= -1.39 | Decrease the protein stability |
| Polyphen-2 | 1 > 0,4 | Probably damaging |
| GVGD | Class 65 | Most likely interfere with the function |
| fathmm | -6,63 < 0 | Damaging |
| I-mutant |  | Decrease stability |
| MetaLR | 0.9878 | Pathogenic |
| EIGEN | 0.744 | Pathogenic |
| FATHMM-MKL | 0.987 | Pathogenic |
| M-CAP | 0.8999 | Pathogenic |
